# RAA-Cas12a-Tg: A Nucleic Acid Detection System for *Toxoplasma gondii* Based on CRISPR-Cas12a Combined with Recombinase-Aided Amplification (RAA)

**DOI:** 10.3390/microorganisms9081644

**Published:** 2021-07-31

**Authors:** Qiao-Ni Ma, Meng Wang, Lai-Bao Zheng, Zi-Qin Lin, Muhammad Ehsan, Xing-Xing Xiao, Xing-Quan Zhu

**Affiliations:** 1 State Key Laboratory of Veterinary Etiological Biology, Key Laboratory of Veterinary Parasitology of Gansu Province, Lanzhou Veterinary Research Institute, Chinese Academy of Agricultural Sciences, Lanzhou 730046, China; maxintan1228@gmail.com (Q.-N.M.); wangmeng02@caas.cn (M.W.); 2 Wenzhou Key Laboratory of Sanitary Microbiology, Key Laboratory of Laboratory Medicine, Ministry of Education, School of Laboratory Medicine and Life Sciences, Wenzhou Medical University, Wenzhou 325035, China; zhenglaibao@wmu.edu.cn (L.-B.Z.); ziqinlin@wmu.edu.cn (Z.-Q.L.); 3 Department of Parasitology, Faculty of Veterinary and Animal Sciences, The Islamia University of Bahawalpur, Bahawalpur 63100, Pakistan; mehsan124@gmail.com; 4 College of Veterinary Medicine, Shanxi Agricultural University, Taigu, Jinzhong 030801, China; 5 Key Laboratory of Veterinary Public Health of Yunnan Province, College of Veterinary Medicine, Yunnan Agricultural University, Kunming 650201, China

**Keywords:** *Toxoplasma gondii*, detection, RAA-Cas12a-Tg system, soil

## Abstract

Toxoplasmosis, caused by the intracellular protozoon *Toxoplasma gondii*, is a significant parasitic zoonosis with a world-wide distribution. As a main transmission route, human infection can be acquired by the ingestion of *T. gondii* oocysts from the environment (e.g., soil, water, fruits and vegetables). Regarding the detection of *T. gondii* oocysts in environmental samples, the development of a time-saving, cost-effective and highly sensitive technique is crucial for the surveillance, prevention and control of toxoplasmosis. In this study, we developed a new method by combining recombinase-aided amplification (RAA) with CRISPR-Cas12a, designated as the RAA-Cas12a-Tg system. Here, we compared this system targeting the 529 bp repeat element (529 bp-RE) with the routine PCR targeting both 529 bp-RE and ITS-1 gene, respectively, to assess its ability to detect *T. gondii* oocysts in soil samples. Our results indicated that the 529 bp RE-based RAA-Cas12a-Tg system was able to detect *T. gondii* successfully in nearly an hour at body temperature and was more sensitive than the routine PCR assay. The sensitivity of this system reached as low as 1 fM with high specificity. Thus, RAA-Cas12a-Tg system provided a rapid, sensitive and easily operable method for point-of-care detection of *T. gondii* oocysts in soil, which will facilitate the control of *T. gondii* infection in humans and animals.

## 1. Introduction

Toxoplasmosis is an important zoonotic disease caused by the protozoon *Toxoplasma gondii*, which is an opportunistic pathogen with the capacity of infecting nearly all warm-blooded animal species and one third of the world’s human populations [1,2]. For example, approximately 10% of Chinese people were infected with *T. gondii* [2]. Without effective treatment and vaccines, infection with *T. gondii* can cause serious clinical symptoms and even fatal consequences in immunocompromised patients (e.g., HIV/ADIS and transplant patients) and congenitally infected infants [3,4]. Toxoplasmosis also can result in the abortion and stillbirth of livestock, causing considerable economic losses to animal husbandry [1,5]. As a foodborne and waterborne pathogen, *T. gondii* is capable of inadvertently appearing in vegetables, fruits, water and soil [6,7,8,9,10,11,12,13]. Human and animal infections occur through eating raw or undercooked meat or via the ingestion of water or food contaminated with sporulated oocysts [14]. Therefore, the rapid, highly specific and accurate detection of *T. gondii* from natural environment is crucial for the surveillance, prevention and control of toxoplasmosis.

Conventional diagnostic methods such as light microscope examination are time-consuming and unreliable, and require a large volume of biological and environmental samples [15]. Serological diagnosis also has some limitations, for example, it is difficult to diagnose toxoplasmosis in immunocompromised patients [16]. However, molecular methods are independent of the host’s immune response, and allow direct detection of the parasite [17]. Currently, molecular methods supporting the *T. gondii* detection are mainly emerging from PCR-based technologies, such as routine PCR, real-time PCR (RT-PCR), and loop-mediated isothermal amplification (LAMP) [15]. For these molecular technologies, *T. gondii*-specific repetitive DNA sequences, such as B1 gene, ITS-1 and 18S rDNA, and 529-bp repeat element (RE), have shown good sensitivities for the detection of *T. gondii* [15]. In contrast, PCR and LAMP assays targeting the 529-bp RE sequence have suggested that they are more sensitive than the B1 gene-based assays [9,18,19], while the multicopy ITS-1 and 18S rDNA have shown a similar sensitivity of the B1 gene [20,21]. In addition to sensitivity evaluation of target molecules, LAMP is more sensitive than the routine PCR, but slightly lower than the RT-PCR [22,23]. Although RT-PCR-based methods now hold well performance especially for detecting low concentration of target DNA, the commercial RT-PCR assay is expensive, instrument-dependent and time-consuming. Therefore, the development of an easy, sensitive and cost-effective molecular method is warranted.

As it has the ability named trans-cleavage to accurately recognize and cleave specific nucleic acid target, CRISPR-Cas systems have shown great potential as practical diagnostic tools [24,25]. The Cas nucleases contain RNA-guided RNases (Cas13a and Cas13b) and RNA-guided DNases (Cas12a, Cas12b and Cas14), which are able to exhibit non-specific trans-cleavage activity after binding to their specific targets and has been fully evaluated for the detection of nucleic acids [26,27,28]. For Cas orthologues, Cas12a and Cas13 are the main nucleases for the development of CRISPR-Cas-based nucleic acid detection method with higher sensitivity and specificity for the capability of recognizing a nucleic acid target, and they also have been developed to combine with isothermal amplification technologies, such as Recombinase-aided Amplification (RAA), to enhance the diagnostic accuracy [29,30,31]. Depending on the type of nucleic acid substrate (DNA or RNA), CRISPR-Cas12a/Cas13-based systems have been alternatively applied to the detection of different pathogens, including viruses [26,29,32,33,34], bacteria [31,35,36,37], parasitic protozoa *Plasmodium* spp. [38] and *Cryptosporidium* spp. [39]. However, to date, there has been no report of the use of CRISPR-Cas system for the detection of *T. gondii* yet.

**Figure 1:**
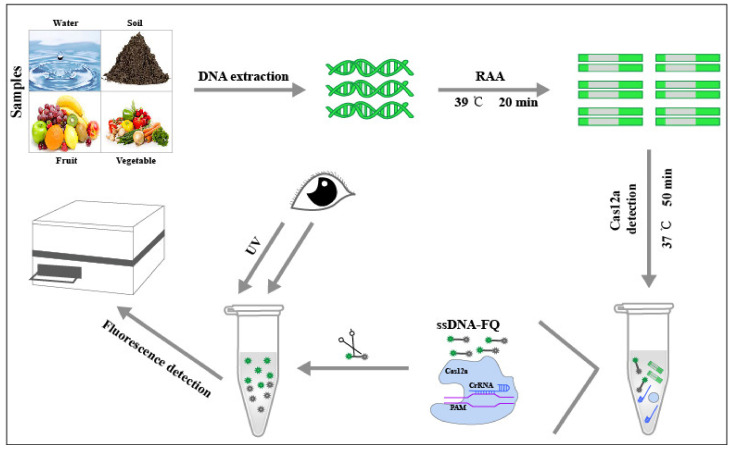
Schematic diagram of the application of the RAA-Cas12a-Tg detection system to environmental samples such as soil, water, fruit and vegetable.

In this work, by designing specific CRISPR-derived RNA (crRNA) probe targeting the 529 bp-RE sequence of *T. gondii* and combining RAA and Cas12a protein, termed RAA-Cas12a-Tg system, we presented an advanced approach for easy, rapid and accurate detection of *T. gondii* DNA by observing the fluorescence intensity with the naked eye under an ultraviolet light or via the detection of the fluorescence wavelength using a microplate reader (Figure 1). Additionally, by examination of 30 soil DNA samples from our previous study [9], we also compared the *T. gondii*-positive results detected by the conventional PCR-based method and RAA-Cas12a-Tg system. Our verification indicated that RAA-Cas12a-Tg system has merits in the aspect of conveniency and sensitivity, and its applications may facilitate the control of toxoplasmosis in humans and animals.

## 2. Materials and Methods

### 2.1. Materials

All primers used for RAA assay and PCR amplifications were synthesized by TSINGKE Biotech (Xi’an, China). ssDNA-FQ was synthesized by Sangon Biotech (Shanghai, China) and crRNA was synthesized by GenePharma (Shanghai, China). The basic RAA kits were purchased from ZC Bio-Sci&Tech (Hangzhou, China). A total of 30 soil DNA samples were provided by Dr. Wei Cong of Marine College, Shandong University, China [9]. Seven parasite DNA samples (*T. gondii*, *Cryptosporidium parvum*, *Neospora caninum*, *Enterocytozoon bieneusi*, *Blastocystis* sp., *Eimeria tenella*, and *Toxocara canis*) were prepared by our lab and stored at −20 °C until for RAA- and PCR-based assays.

### 2.2. Establishment of 529 bp RE-Based RAA Assay

Four pairs of RAA primers were designed using Primer Premier 5 software [40]. The primer sequences and lengths are summarized in Table 1. The nucleotide sequence of *T. gondii* 529 bp-RE (RH strain) was downloaded from NCBI GenBank (https://www.ncbi.nlm.nih.gov/genbank/, accessed on 20 November 2020) with accession number AF146527.1 [18]. The obtained DNA of each sample was used for RAA reaction using RAA kit assay. Using 2 μL of DNA template, a total 50 μL reaction system was performed in a 200 μL aseptic single tube that contained lyophilized powder, with 41.5 μL of buffer A, 2 μL each of forward and reverse primers and 2.5 μL of buffer B. The reaction tube was inverted 6~8 times and then centrifuged for 15 s. The reaction tube was placed on a Thermostatic Water Bath, and then incubated at 39 °C for 20 min. The amplification products were purified using Universal DNA Purification Kit (Tiangen, Beijing, China).

### 2.3. Construction of Positive Recombinant Plasmids pMD18-T-529 bp

As shown in Table 1, we first designed a pair of plasmid primer based on the 529 bp-RE sequence using Primer Premier 5 software in order to establish the RAA-Cas12a-Tg system and to evaluate the sensitivity of this method. The 25 μL PCR reaction system contained 12.5 μL Pre-Mix Taq Enzyme, 1.5 μL each of forward and reverse primers, 2 μL DNA template, and 7.5 μL ddH_2_O. The PCR was performed under the following cycling conditions: initial denaturation at 94 °C for 5 min, followed by 34 cycles of 94 °C for 30 s, 57 °C for 30 s and 72 °C for 1 min, with an additional 5 min final extension at 72 °C. PCR products were examined by 1% agarose gel electrophoresis and then were recovered using E.Z.N.A^TM^ Plasmid Mini Kit (OMEGA Bio-Tek, Norcross, GA, USA). Then, the gel recovered products were cloned into the pMD^TM^ 18-T vector (Takara, Shanghai, China). Finally, the concentration of positive recombinant plasmids was qualified using with the NanoPhotometer (IMPLEN, Munich, Germany), and the recombinant plasmid concentration was diluted from 10^2^ to 10^−7^ nM, and then stored at −20 °C for the subsequent fluorescent detection of the Cas12a assay.

### 2.4. crRNA/ssDNA-FQ Preparation and Fluorescence Detection of Cas12a

The CRISPR-derived RNA (crRNA) and ssDNA-FQ sequences, as shown in Table 1, were designed based on Cas12a-mediated fluorescence detection reported by Zhang et al. [41]. crRNA, ssDNA-FQ reporter, EnGen Lba Cas12a protein (NEB, Ipswich, MA, USA), 10x NEBuffer 2.0 (NEB, Ipswich, MA, USA) and positive recombinant plasmids pMD^TM^ 18-T-529-bp (Takara, Shanghai, China) were used for Cas12a-mediated fluorescence detection. The processes of fluorescence detection was performed under the following procedures: (i) 800 nM Cas12a and 1 μM crRNA each of 20 μL were simultaneously added to a 200 μL aseptic PCR tube, and then incubated at 37 °C for 20 min; (ii) recombinant plasmids and 1 μM ssDNA-FQ reporter each of 20 μL were simultaneously added to the PCR tube of first-step, and then incubated at 37 °C for 30 min; (iii) after the aforementioned reaction, the final products were transferred into a black 96-well plate to detect the fluorescence with an excitation wavelength of 490 nm, and the fluorescence was detected at wavelength of 510 nm to 600 nm using the Varioskan™ LUX microplate reader (Thermo Scientific, Waltham, MA, USA). During all the procedures, the concentration of crRNA, ssDNA-FQ reporter, Cas12a Protein and recombinant plasmids were diluted using 1x NEBuffer 2.0 (NEB, Ipswich, MA, USA) according to the actual requirements.

### 2.5. Analysis of Specificity and Sensitivity of RAA-Cas12a System

Each of 2 μL DNA templates from *T. gondii* and the six “control” parasites were used for RAA reaction, respectively, and then we tested the specificity of RAA-Cas2a-Tg system by comparing *T. gondii* targeting the 529 bp-RE with other parasite DNA templates targeting the 529 bp-RE by Cas12a-mediated fluorescence detection. In terms of sensitivity detection experiments, 10-fold serial dilutions ranging from 10^2^ to 10^−7^ nM of the positive recombinant plasmids were utilized, and by mean of Student’s *t*-test we also compared the detection sensitivity differences between different diluted plasmid DNA concentrations.

### 2.6. Application of 529 bp RE-Based RAA-Cas12a System Detection to Soil Samples

The performance of 529 bp RE-based RAA-Cas12a-Tg system was evaluated using 30 environmental soil DNA samples. Briefly, 2 μL of soil DNA samples were used for RAA reaction, and the procedure was completed in 20 min; 20 μL of RAA amplification product was added to the Cas12a-mediated fluorescence system and the fluorescence detection was completed in 50 min. We compared the detected results between RAA-Cas12a-Tg system and conventional PCR-based detection, and positive PCR products were sent to TSINGKE Biotech (Xi’an, China) for DNA sequencing.

## 3. Results

### 3.1. Optimization of the RAA System

**Figure 2:**
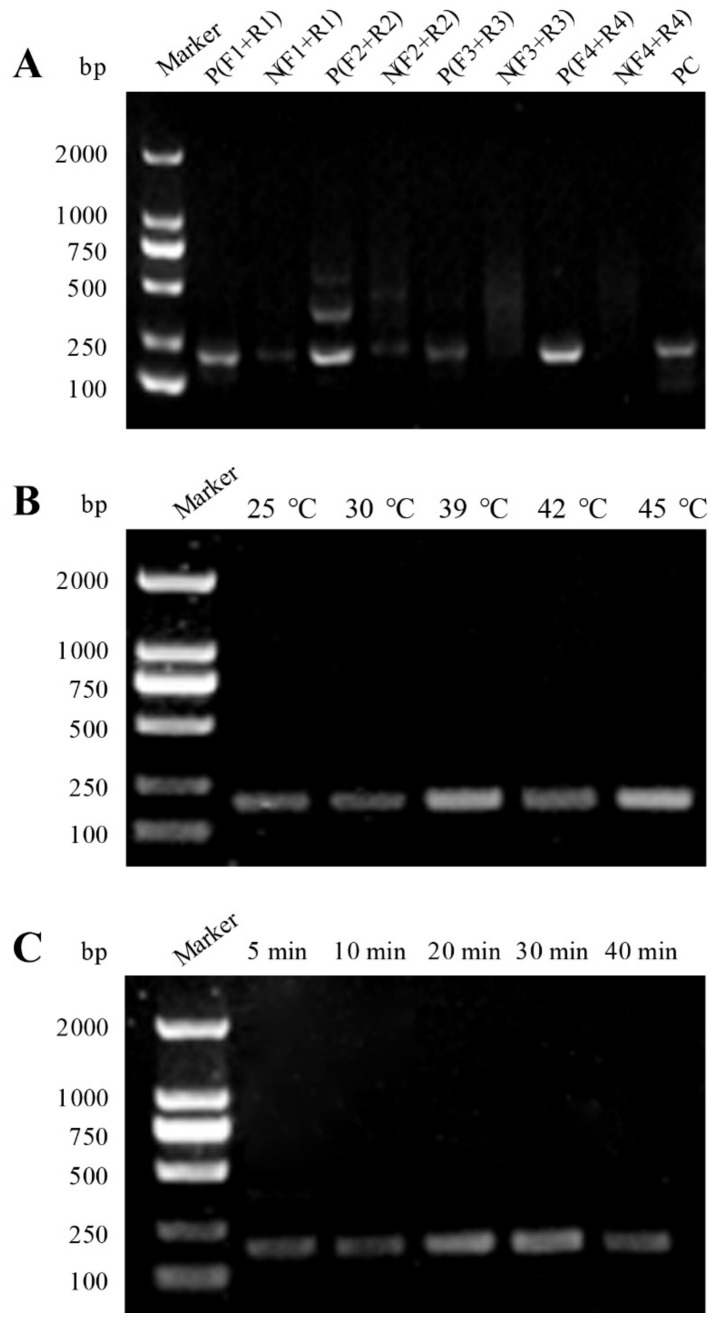
Optimization of the RAA assay. (**A**) Screening of the RAA primers. Agarose gel electrophoresis of RAA amplification products using different primer combinations. (**B**) Optimization of reaction temperature of the RAA assay. (**C**) Optimization of reaction time of the RAA assay.

Four pairs of 529 bp RE-based RAA primers (F1/R1, F2/R2, F3/R3 and F4/R4) were designed and, as shown in Table 1, these primers were identified by RAA amplification, respectively. By comparison, the agarose gel electrophoresis (AGE) results showed that the P(F4 + R4) primer combination had a relatively specific band and high intensity at 186 bp, and the negative group N(F4 + R4) did not present any band (Figure 2A). Thus, the P(F4 + R4) primer combination was considered as the optimal primer pair for RAA system optimization. With regard to the RAA reaction temperature, five gradients including 25, 30, 39, 42, and 45 °C were chosen for RAA reaction, and all of them were able to amplify a clear band with 186 bp size in the AGE results (Figure 2B). In addition, we chose 39 °C as the optimal temperature to determine the RAA reaction time and found that the DNA targeting 529 bp-RE could result in a clear band at 186 bp at five different time points (i.e., 5, 10, 20, 30 and 40 min) (Figure 2C). Thus, RAA reaction at 20 min was used for further experiment.

### 3.2. Optimization of Cas12a-Mediated Fluorescence Detection Assay

**Figure 3:**
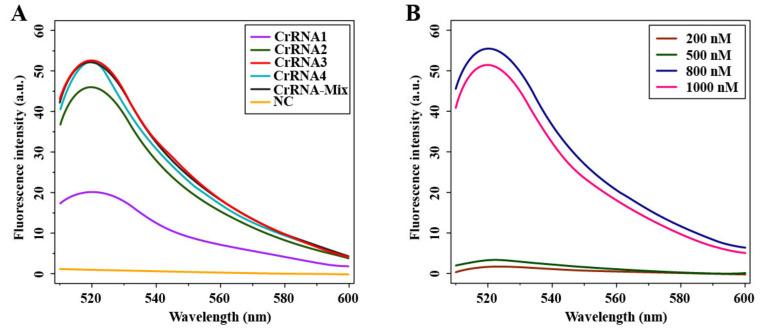
Optimization of the fluorescence detection of Cas12a. (**A**) Screening of the crRNA sequence. crRNA1, crRNA2, crRNA3 and crRNA4 represent different crRNAs; crRNA-MIX represents the product of the above four crRNAs mixed in the same proportion; NC stands for negative control. (**B**) Optimization of the concentration of LbCas12a.

Four crRNA sequences (crRNA1, crRNA2, crRNA3 and crRNA4) were designed and prepared (Table 1), and we used the Cas12a-mediated fluorescence system to detect the crRNA-guided Cas12a cleavage activity and to select a suitable crRNA for further experiment. Within the emission wavelength of 510 to 600 nm, as shown in Figure 3A, all four crRNAs and the crRNA-Mix (mixed in equal proportions for these crRNA) had a fluorescence intensity and reached the highest fluorescence value (plateau phase) at 520 nm. From this result, we found that crRNA3 illustrated a better curve performance within the whole emission wavelength, and therefore this crRNA was considered as the best one for the Cas12a-mediated fluorescence detection assay. Additionally, we optimized the concentration of Cas12a protein in our Cas12a-mediated fluorescence detection assay, as the Cas12a concentration plays a crucial role in the detection of fluorescence. Four different concentration gradient values of Cas12a protein, including 200, 500, 800 and 1000 nM, were tested in this study. As shown in Figure 3B, the results showed that all concentration values had fluorescence signal and, relatively, the concentration at 800 nM had much higher fluorescence intensity than that of other three concentration values (i.e., 200, 500 and 1000 nM) within the emission wavelength of 510 to 600 nm. Thus, the concentration at 800 nM was chosen as the optimal reaction value for Case12a protein.

### 3.3. Evaluation of Sensitivity and Specificity of the RAA-Cas12a-Tg System

**Figure 4:**
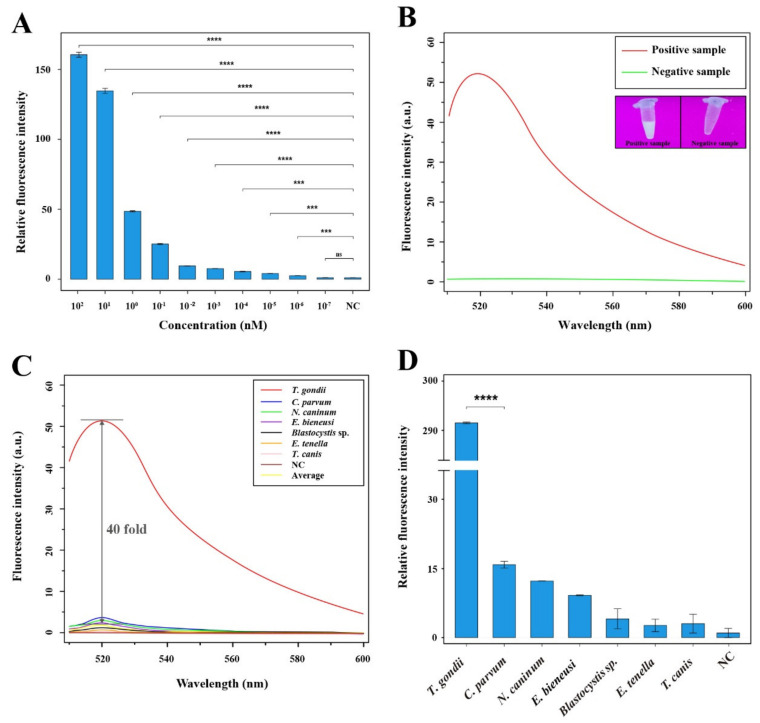
Analysis of specificity and sensitivity of the RAA-Cas12a-Tg system. (**A**) Sensitivity of the RAA-Cas12a system for *T. gondii* detection. Relative fluorescence intensity was estimated by the formula of (Ft-F0)/(Fn-F0) × 100%, where Ft, F0, and Fn represent the fluorescence peak values of the positive recombinant pMD18-T-529 bp plasmids after dilution by 10-fold serial ranging from 10^2^ to 10^−7^ nM, blank, and negative control, respectively. Error bars represent the mean standard deviation (SD), where *n* = 2 replicates. **** *p* ≤ 0.0001; *** *p* ≤ 0.001; ns *p* > 0.05. (**B**) Visual detection of signal amplification by the naked eye under a UV transilluminator and by an observation of microplate reader of the positive sample containing *T. gondii* DNA and the negative sample without *T. gondii* DNA. (**C**) Specificity of the RAA-Cas12a system for *T. gondii* detection. The nucleic acids of other six parasites including *C. parvum*, *N. caninum*, *E. bieneusi*, *Blastocystis* sp., *E. tenella* and *T. canis* were used to evaluate the specificity of the RAA-Cas12a-Tg system. Average represents the largest fluorescence intensity of other six parasites at plateau phase; NC stands for negative control. (**D**) Specificity of the RAA-Cas12a system for *T. gondii* detection. The relative fluorescence intensity of *T. gondii* detection using the RAA-Cas12a-Tg system was more significant than that of the closely related *C. parvum* and other five parasites. Error bars represent the mean SD, where *n* = 2 replicates. **** *p* ≤ 0.0001.

To evaluate the sensitivity of the RAA-Cas12a system, the positive recombinant pMD18-T-529 bp plasmids diluted by 10-fold serial (ranging from 10^2^ to 10^−7^ nM) were used for fluorescence intensity test at excitation wavelength of 510 nm to 600 nm. The statistical analysis revealed that the plasmids ranging from 10^2^ to 10^−6^ nM had significantly higher fluorescence intensity than that of the negative control group (**** *p* < 0.0001 and *** *p* < 0.001); however, there was no significant difference between the group with 10^−7^ nM and NC group (ns *p* > 0.05) (Figure 4A). Visual detection of signal amplification demonstrated that the positive sample containing *T. gondii* DNA had an obvious signal by the naked eye under a UV transilluminator and bear a maximum test-band fluorescence intensity at the plateau phase (520 nm) by observation using a microplate reader, while the negative sample without *T. gondii* DNA did not exhibit any signal amplification using these two methods (Figure 4B).

We also evaluated the specificity by experimentally detecting and comparing the fluorescence intensity for *T. gondii* and other selected control parasites (*C. parvum*, *N. caninum*, *E. bieneusi*, *Blastocystis* sp., *E. tenella* and *T. canis*). As shown in Figure 4C, the results showed that fluorescence signal for *T. gondii* detection was significantly higher than that of other parasites and the NC group, and the largest fluorescence intensity fold between *T. gondii* and other parasites (average value) at plateau phase was able to reach 40-fold. Additionally, analysis of relative fluorescence intensity showed that RAA-Cas12a-Tg system for *T. gondii* detection was more significant than that of the genetically closely and distant parasites (**** *p* < 0.0001) (Figure 4D). These results suggested that the RAA-Cas12a system for *T. gondii* detection had sufficient specificity.

### 3.4. Application of the 529 bp RE-Based RAA-Cas12a-Tg System for T. gondii Detection in Soil Samples

**Figure 5:**
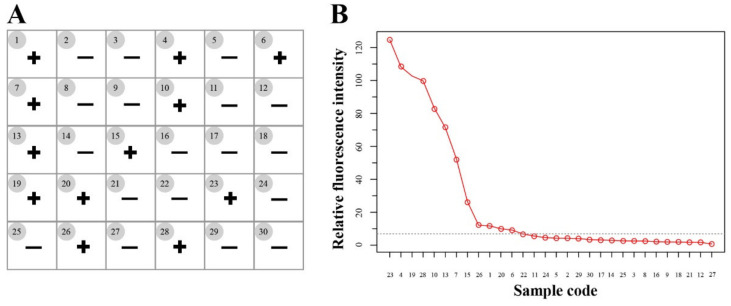
Application of the RAA-Cas12a-Tg system to *T. gondii* detection in the environment samples. (**A**) Summary of *T. gondii* detection in soil samples (*n* = 30) using the RAA-Cas12a-Tg system. + means positive and – means negative. (**B**) 30 soil samples with relative fluorescence intensity > 9 were considered as positive.

We examined 30 soil DNA samples using the RAA-Cas12a-Tg system, and samples with relative fluorescence intensity were further evaluated by DNA sequencing. The results showed that 12 out of the 30 soil DNA samples were *T. gondii* positive detected by the RAA-Cas12a-Tg system (Figure 5A) and samples with relative fluorescence intensity > 9 were considered positive according to the result of sequencing (Figure 5B), while 10 samples were positive for *T. gondii* by conventional PCR method targeting both the 529-RE sequence and the ITS-1 rDNA [9], which was 100% consistent with 10 out of the 12 positive samples by our RAA-Cas12a-Tg system. Meanwhile, all positive samples were sequenced by TSINGKE Biotech (Xi’an, China), and the results showed that all the sequences represented the 529 bp repeat element of *T. gondii*. Thus, the RAA-Cas12a-Tg system were more sensitive than the conventional PCR method.

## 4. Discussion

Rapid and accurate detection of potential *T. gondii* DNA in the environment plays an important role in identifying, assessing and managing health and safety risks caused by the parasite, especially for emerging public health problems linked to food-, water- and soil-borne outbreaks [9,12,42,43,44,45]. In the present study, we established a technique, designated as RAA-Cas12a-Tg system, for the detection of *T. gondii* DNA in soil. This system does not require an incubator higher than 39 °C, and its reaction can even be completed at the temperature of ~37 °C. The whole reaction comprises test reagents that can be easily stored in a low-temperature vessel, and without the need of sophisticated technical requirements (Figure 1). The whole reaction process used in the study takes nearly 1 h, and the results for highly copied nucleic acid can be easily obtained by ultraviolet irradiation with the naked eye (Figure 4B), or a handheld microplate reader device [46]. Therefore, the RAA-Cas12a-Tg system is particularly suitable for an on-site *T. gondii* detection (e.g., sewage plant, park or farm) in a short time and without the requirement of an available diagnostic laboratory.

The option of an appropriate nucleic acid-amplified technology to combine with the CRISPR-Ca12a system plays a critical role in the clinical diagnosis. The common PCR-based technologies such as routine PCR and RT-PCR amplifications require a thermal cycle (usually 95-60-72 °C) for more than 2 h, while LAMP amplification requires a constant temperature of ~65 °C for more than 1 h, and the primer design of LAMP assay is cumbersome. Thus, PCR, RT-PCR and LAMP technologies combining a CRISPR-Ca12a system are bulky and not suitable for rapid diagnosis. Furthermore, the Cas12a assay needs to be carried out at a temperature below 45 °C, otherwise the Cas12a nuclease would appear irreversible damage thereby affecting the cleavage rate of the CRISPR system [30,47]. Hence, if a single-tube one-step reaction is performed, PCR, RT-PCR and LAMP technologies are also not suitable for combination with a CRISPR-Ca12a system. In the present study, the RAA system is a good option as the initial signal amplification, because RAA assay is much cheaper, just takes 20 min or lesser time, and the required temperature (37~45 °C) is consistent with the optional temperature of CRISPR-Ca12a system. Additionally, we also optimized the RAA system by testing primers, reaction temperatures and times (Figure 2A–C), which ensures the RAA reaction specificity and provides alternative diagnostic conditions in practical applications.

The RAA-Cas12a-Tg system herein developed in this study is an ultrasensitive and robust platform for detection of pathogens. Some recent studies have verified that the signal amplification effect of Cas12a protein and the amplification procedure could make the fluorescence readout increase exponentially [48,49], and the sensitivity of our method is at femtomolar level (Figure 4A). Additionally, our method has the high specificity that mainly depends on the specific recognition of LbCas12a-crRNA targeting the single nucleotide mutations [50]. Compared with the conventional methods, our method is more sensitive and appropriate for point-of-care detection of environment samples without the need of sophisticated equipment. Therefore, this system can be used in laboratories with poor conditions or in the field.

While this new system has some advantages mentioned above, it still has some limitations. During the two separate processes, the potential cross contamination can be caused, thus the future research should mainly focus on the development of single-tube one-step detection which requires less time and more accuracy. Besides, the concentration of Cas12a used in this study were high, which increased the cost slightly. However, 200 nM Cas12a protein still could work but the result was not as good as that of 800 nM Cas12a. Nevertheless, this novel method is a new attempt to detect *T. gondii* in the environment, and it also can be applied to the detection of other pathogens.

## 5. Conclusions

RAA-Cas12a assay is a promising detection technology that is rapid, reliable, ultrasensitive and requiring no expensive instruments. Most importantly, this method can be performed at a low temperature environment, which makes it a practical on-site detection method to detect *T. gondii* in the field using a portable thermostatic heater and handheld fluorescence detector.

## Figures and Tables

**Table 1:** Primer sequences for RAA, plasmid, crRNA and ssDNA-FQ.

Assay	Primer Name	Sequence (5′→3′)	Product Size (bp)
RAA	529bp-RAA-F1	GAAGGGACAGAAGTCGAAGGGGA	171
	529bp-RAA-R1	GAAAAGCAGCCAAGCCGGAAACA	
	529bp-RAA-F2	TGGAGCCACAGAAGGGACAGAAGT	184
	529bp-RAA-R2	CAGGAAAAGCAGCCAAGCCGGAAA	
	529bp-RAA-F3	CAGAAGGGACAGAAGTCGAAGGGGA	175
	529bp-RAA-R3	AGGAAAAGCAGCCAAGCCGGAAACA	
	529bp-RAA-F4	GAGCCACAGAAGGGACAGAAGTCG	186
	529bp-RAA-R4	CCTCCAGGAAAAGCAGCCAAGCCG	
Plasmid	529bp-PF	GGAGGAAGACGAAAGTTG	515
	529bp-PR	ACAGTGCATCTGGATTCC	
crRNA	crRNA1	UAAUUUCUACUAAGUGUAGAUACTCGGGCCCAGCTGCGTCT	
	crRNA2	UAAUUUCUACUAAGUGUAGAUACAGGCAAGCTCGCCTGTGC	
	crRNA3	UAAUUUCUACUAAGUGUAGAUCACCCUCCAGGAAAAGCAGCCA	
	crRNA4	UAAUUUCUACUAAGUGUAGAUCTCGTGGTGATGGCGGAGAG	
ssDNA-FQ	TgCas12a	6FAM-CCGGAAAAAAAAAAAACCGG-BHQ1	

## Data Availability

The data that support the figures within this paper and other findings of this study are available from the corresponding authors upon reasonable request.
